# Supplementary figures and images for: mRNA sequencing reveals the distinct gene expression and biological functions in cardiac fibroblasts regulated by recombinant fibroblast growth factor 2
Source: PeerJ. 2023 Jul 19;11:e15736. doi: 10.7717/peerj.15736 (PMC10362857; doi:10.7717/peerj.15736)

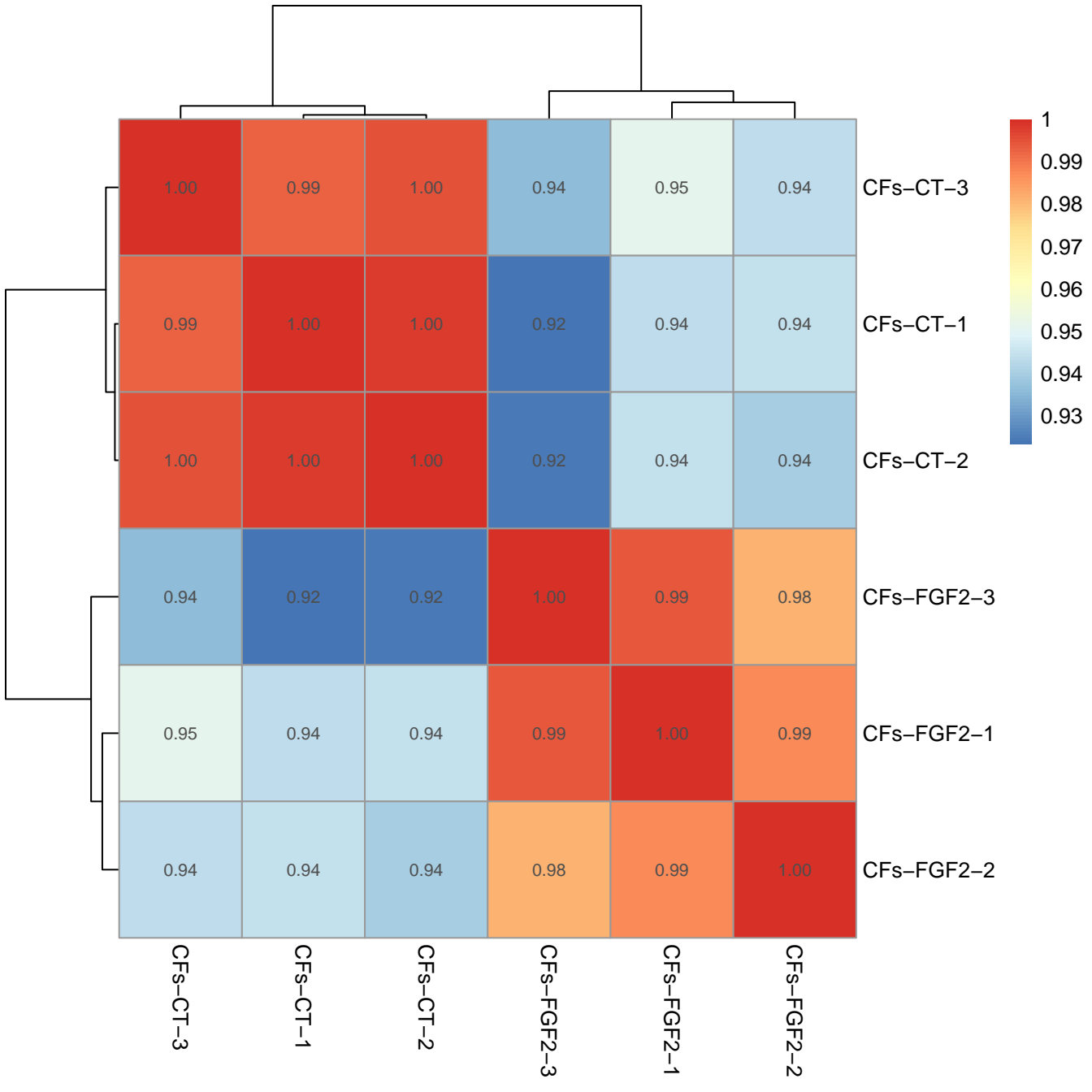

Supplement: Supplemental Information 1 — The values of Pearson correlation Coefficient are displayed. CFs-CT: CFs-CON; CFs-FGF2: CFs-FGF2. [file peerj-11-15736-s001.pdf]

**(A)**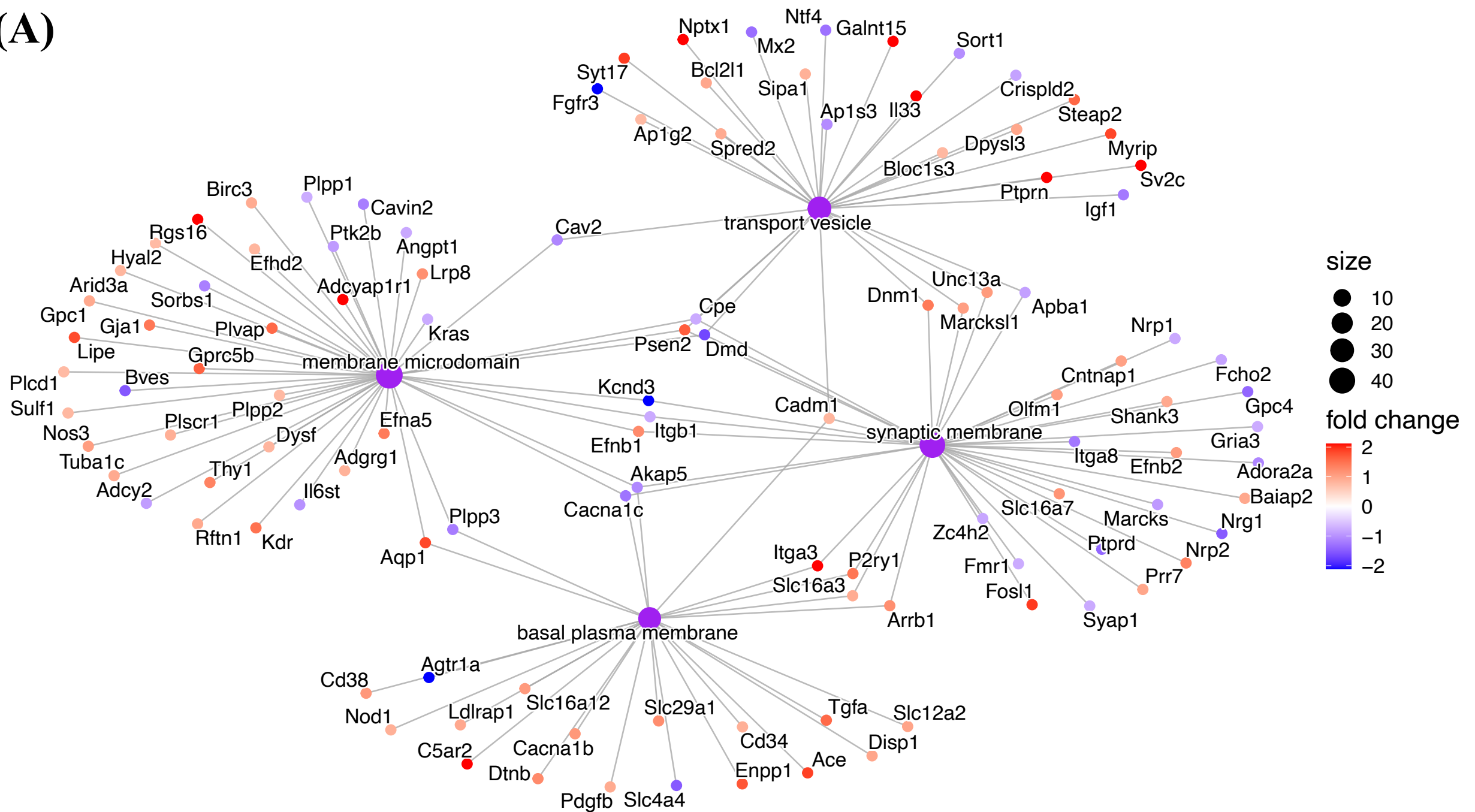**(B)**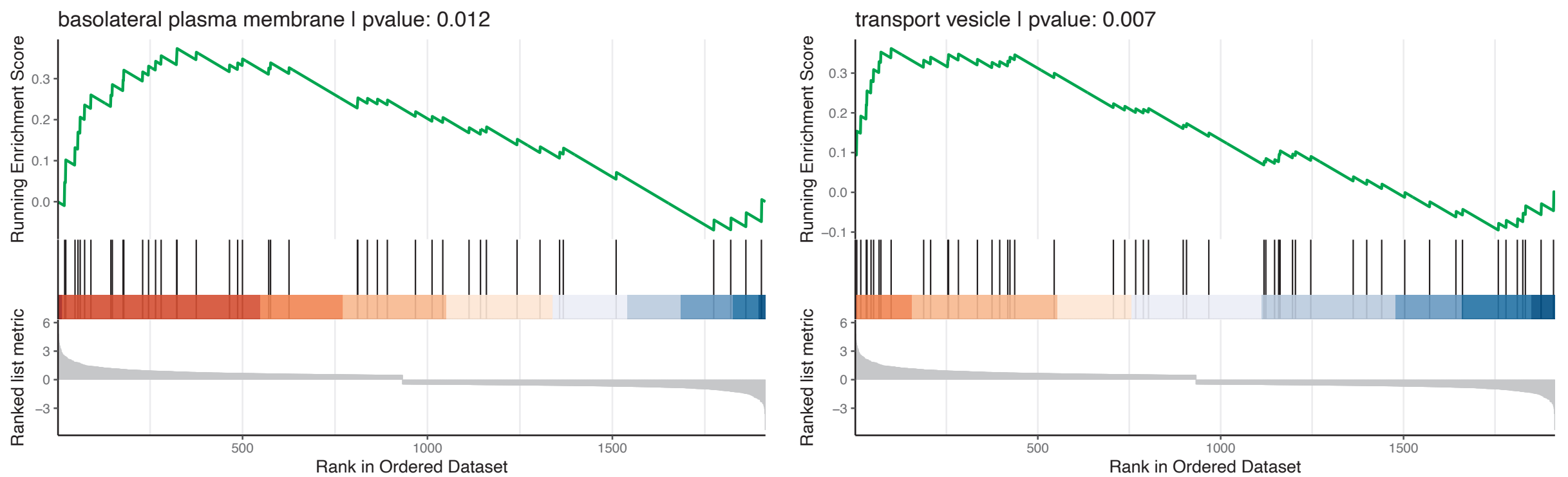

Supplement: Supplemental Information 2 — (A): Display of genes related to membrane microdomain, basolateral plasma membrane, Golgi membrane, transport vesicle and synaptic membrane. (B): GSEA plots of basolateral plasma membrane and transport vesicle functions. [file peerj-11-15736-s002.pdf]

**(A)**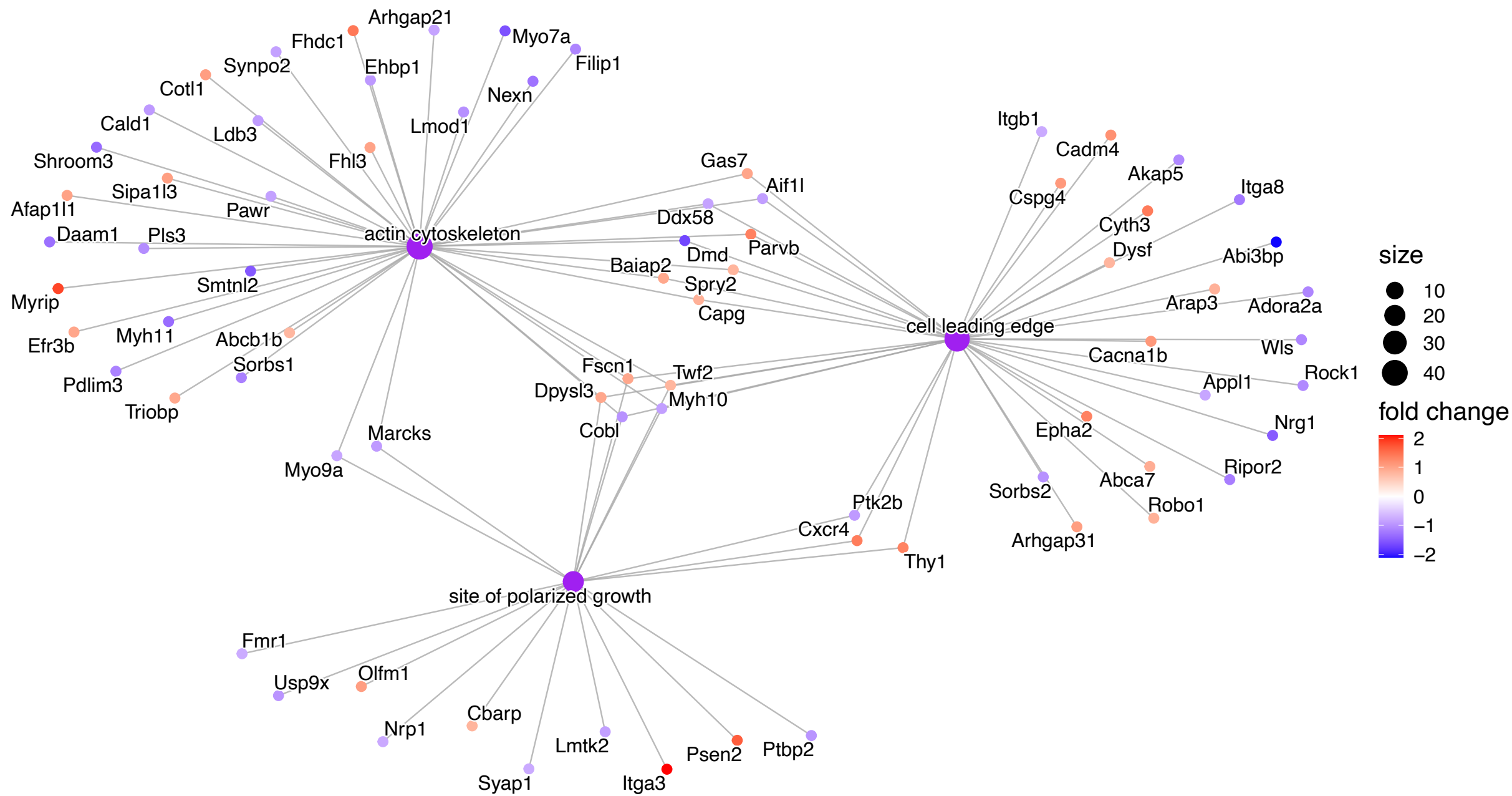**(B)**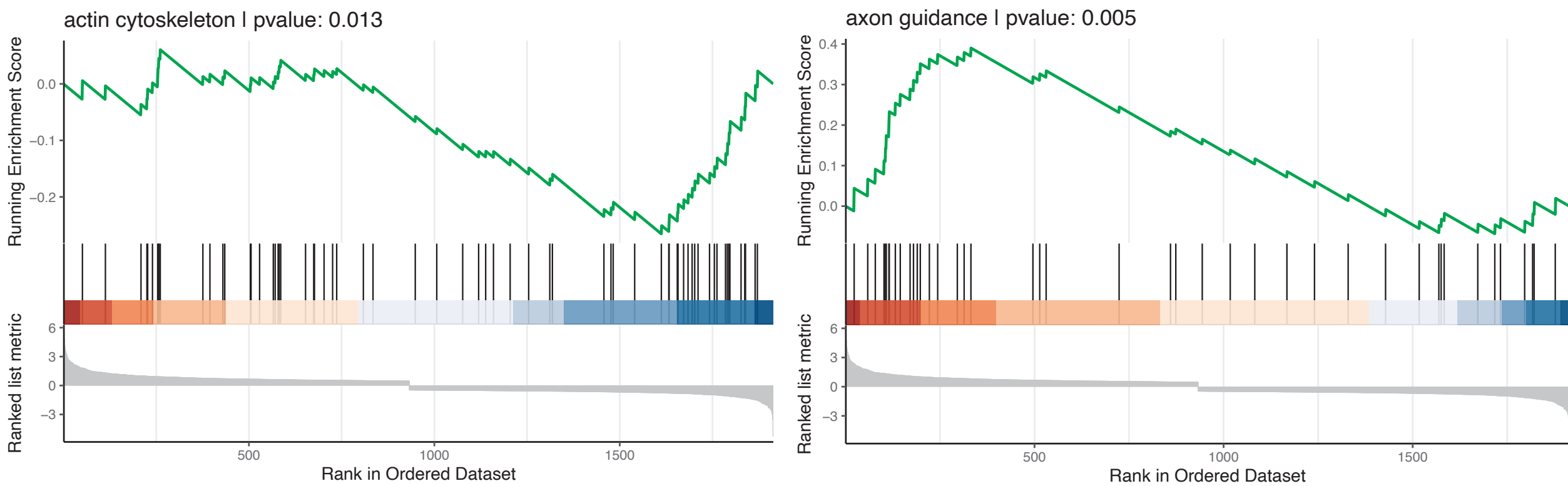

Supplement: Supplemental Information 3 — (A): Display of genes related to actin cytoskeleton, apical part of cell, cell leading edge and site of polarized growth. (B): GSEA plots of actin cytoskeleton and axon guidance. [file peerj-11-15736-s003.pdf]
