# Supplementary material for: mRNA sequencing reveals the distinct gene expression and biological functions in cardiac fibroblasts regulated by recombinant fibroblast growth factor 2
Source: PeerJ. 2023 Jul 19;11:e15736. doi: 10.7717/peerj.15736 (PMC10362857; doi:10.7717/peerj.15736)
Supplement: Supplemental Information 4 [file peerj-11-15736-s004.pdf]

Table S1. Primers for RT-PCR in this study.

Primer	Oligonucleotide Sequences (5'-3')
Col3a1F	GGCCTCCCAGAACATTACATACC
Col3a1R	CATGGCCTTGCGTGTTTG
ElnF	AAACTGCCCTATGGAGTGGC
ElnR	TCCAGCACCATACTTCGCTG
ItgblF	TTGATCCAACTGGCGACTGG
ItgblR	TGGGTGCTCACACTTCTTCC
Acta2F	CATCACCAACTGGGACGACA
Acta2R	TCCGTTAGCAAGGTCGGATG
Thbs2F	CCACCAGAACAACCAAGACAAC
Thbs2R	ATCATCGGAGTCACAGGCATC
GAPDHF	ACACCCACTCCTCCACCTTT
GAPDHR	TTACTCCTTGGAGGCCATGT
Ccdc80F	GGATGACGAGGACTTGGTAGAC
Ccdc80R	AACACGGACTTCATTGCTATTGG
TaglnF	TGGCTGAAGAATGGCGTGAT
TaglnR	TCCATCGTTCTTGGTCACGG
Gas6F	TAGAAGTCGTGGCTCGCATT
Gas6R	GTACCATCCACTTCCAGGGTG
