# Supplementary material for: mRNA sequencing reveals the distinct gene expression and biological functions in cardiac fibroblasts regulated by recombinant fibroblast growth factor 2
Source: PeerJ. 2023 Jul 19;11:e15736. doi: 10.7717/peerj.15736 (PMC10362857; doi:10.7717/peerj.15736)
Supplement: Supplemental Information 5 [file peerj-11-15736-s005.pdf]

Table S2. Differentially expressed genes.

gene_id	baseMean	log2(FoldChange)	GeneSymbol
ENSRNOG00000016388	242.706389	-5.2978738	Sphkap
ENSRNOG00000012031	127.019435	-3.606805	St8sia2
ENSRNOG00000029911	129.487806	-3.0878943	Cilp
ENSRNOG00000016818	1087.01095	-2.8875318	Fgfr3
ENSRNOG00000003587	1326.32653	-2.7352065	Vegfd
ENSRNOG00000017672	115.291994	-2.5754164	Akr1c14
ENSRNOG00000058609	202.829209	-2.5581519	Palmd
ENSRNOG00000005998	390.554909	-2.5324125	Smoc1
ENSRNOG00000023148	1966.54057	-2.4010893	Col11a1
ENSRNOG00000004197	163.96304	-2.382929	Asb12
ENSRNOG00000018346	1049.32297	-2.3667479	Agtr1a
ENSRNOG00000014886	777.527698	-2.366262	Fam107b
ENSRNOG00000025624	189.733912	-2.3436623	Arhgap20
ENSRNOG00000014686	807.878056	-2.2963836	Kcnd3
ENSRNOG00000013166	795.080144	-2.1325899	Wnt4
ENSRNOG00000008336	7984.7563	-2.1181502	Tnfrsf11b
ENSRNOG00000011946	1875.23299	-2.1106572	Ptn
ENSRNOG00000001627	493.316105	-2.0869472	Abi3bp
ENSRNOG00000006670	151.654277	-2.0682362	Rai2
ENSRNOG00000004516	238.054299	-2.061043	Itgbl1
ENSRNOG00000007443	2794.76645	-2.0601423	Jag1
ENSRNOG00000010079	221.929337	-2.0498441	Car3
ENSRNOG00000001469	15449.9402	-2.0022692	Eln
ENSRNOG00000030210	1122.69929	-1.9732143	Fndc1
ENSRNOG00000024602	529.802736	-1.9716946	Plekha7
ENSRNOG00000005094	602.168329	-1.9474594	C1qtnf7
ENSRNOG00000025184	461.856878	-1.9251484	Prss35
ENSRNOG00000007540	337.265456	-1.9223106	Msc
ENSRNOG00000012442	13226.3363	-1.9112155	Cemip
ENSRNOG00000014143	113.783231	-1.900848	Col24a1
ENSRNOG00000019184	190.79089	-1.8800029	Npr3
ENSRNOG00000032659	134.008336	-1.8623229	Plcl1
ENSRNOG00000000875	3234.75311	-1.7837885	Fhl1
ENSRNOG00000014549	218.40339	-1.7707121	Arhgef26
ENSRNOG00000028624	253.297309	-1.759217	Kif26b
ENSRNOG00000029148	427.629457	-1.7391265	Pdgfd
ENSRNOG00000001227	1602.25125	-1.6960397	Adarb1
ENSRNOG00000013072	162.171777	-1.6917659	Plxna4
ENSRNOG00000046366	704.028839	-1.6688362	Dmd
ENSRNOG00000020585	203.242508	-1.6562802	Tbxa2r

ENSRNOG00000003183	921.811105	-1.6405088	Fmod
ENSRNOG00000039902	1527.40241	-1.6097362	Lbh
ENSRNOG00000001645	3416.40286	-1.5907594	Filip1l
ENSRNOG00000013641	154.439706	-1.5823537	Myo7a
ENSRNOG00000016219	434.405484	-1.5816756	Vnn1
ENSRNOG00000011416	1378.61879	-1.5736316	Vegfc
ENSRNOG00000012302	730.350892	-1.5567906	Gucy1a3
ENSRNOG00000002053	262.034418	-1.5563333	Fras1
ENSRNOG00000010666	4118.72815	-1.5328384	Wisp2
ENSRNOG00000043199	121.781185	-1.5250781	Bves
ENSRNOG00000054212	280.103743	-1.52095	Pde1a
ENSRNOG00000015157	127.846087	-1.5208939	Smtnl2
ENSRNOG00000010350	147.226551	-1.513447	Rcan2
ENSRNOG00000017164	1759.0546	-1.503576	Afap1l2
ENSRNOG00000010392	268.437798	-1.5015371	Nrg1
ENSRNOG00000003134	307.645182	-1.5009553	Slc4a4
ENSRNOG00000016213	1028.42204	-1.4975917	Ssbp2
ENSRNOG00000033528	413.211238	-1.4903918	Tll1
ENSRNOG00000002773	679.478892	-1.4670813	Rgs4
ENSRNOG00000008666	338.126311	-1.4610107	Etl4
ENSRNOG00000061910	5012.92908	-1.4598249	Igfbp3
ENSRNOG00000015318	932.034636	-1.4448316	Heyl
ENSRNOG00000048478	417.757192	-1.4443061	Kcne4
ENSRNOG00000002052	44127.6069	-1.4389776	Ccdc80
ENSRNOG00000013364	168.582888	-1.4170435	Hey2
ENSRNOG00000029394	129.995053	-1.4119187	Dusp8
ENSRNOG00000006931	252.399807	-1.3977336	Eepd1
ENSRNOG00000033697	780.402728	-1.3948614	Casp4
ENSRNOG00000002413	5545.24717	-1.3844754	Gpc4
ENSRNOG00000005711	826.503458	-1.3831589	Ptprd
ENSRNOG00000013011	1000.27393	-1.3764733	Dnajb4
ENSRNOG00000018551	380.988603	-1.3752292	Olr63
ENSRNOG00000002097	2769.34168	-1.3739097	Rasl11b
ENSRNOG00000030715	179.533507	-1.3678917	Cfh
ENSRNOG00000014548	1070.88831	-1.3609564	Nedd9
ENSRNOG00000027491	2720.69862	-1.3584024	Vldlr
ENSRNOG00000052444	439.647799	-1.3506304	Samd9
ENSRNOG00000002208	193.908573	-1.3502885	Shroom3
ENSRNOG00000009867	12932.5845	-1.3442072	Tgfb3
ENSRNOG00000057880	3479.88108	-1.3324142	Myh11
ENSRNOG00000015075	159.551461	-1.3314949	Stc1
ENSRNOG00000016012	1442.85393	-1.3266026	Spats2l
ENSRNOG00000001823	326.748809	-1.3226263	St6gal1

ENSRNOG00000001963	1487.22015	-1.3219254	Mx2
ENSRNOG00000012563	2805.72556	-1.3204771	Arhgap29
ENSRNOG00000046660	1233.20413	-1.3194159	Gpc6
ENSRNOG00000016516	114.108392	-1.3125761	Mbp
ENSRNOG00000012367	304.539701	-1.3042992	Pcdh7
ENSRNOG00000033434	446.799408	-1.3029352	Casp12
ENSRNOG00000004345	1178.60627	-1.2996811	Daam1
ENSRNOG00000020783	101.433118	-1.2883335	Ntf4
ENSRNOG00000003745	249.887334	-1.2852952	Atf3
ENSRNOG00000000413	204.984395	-1.2843643	Pln
ENSRNOG00000007964	266.616465	-1.2818889	Tp53inp1
ENSRNOG00000004210	296.735672	-1.2741423	Osr1
ENSRNOG00000012512	548.831752	-1.2708822	Nexn
ENSRNOG00000007090	476.242453	-1.2673379	Cacna1c
ENSRNOG00000019822	940.209444	-1.2577461	Gadd45b
ENSRNOG00000019050	154.93174	-1.256644	Ifit1
ENSRNOG00000002418	3573.0008	-1.2498727	Tgfb2
ENSRNOG00000006545	1544.46611	-1.2381424	44081
ENSRNOG00000048315	916.237487	-1.2281879	Eif2ak2
ENSRNOG00000018865	2229.83561	-1.2240093	Adamts12
ENSRNOG00000033527	124.185276	-1.2225543	Pappa1
ENSRNOG00000016538	2914.16318	-1.2223593	Itga8
ENSRNOG00000004624	1877.10513	-1.2220259	Rnd3
ENSRNOG00000004517	344.944204	-1.2180529	Igf1
ENSRNOG00000015353	124.020527	-1.2103268	Prss12
ENSRNOG00000008622	132.97264	-1.2085908	Creb5
ENSRNOG00000056562	448.545502	-1.2052951	Olfml1
ENSRNOG00000005650	433.89988	-1.2049899	Pgf
ENSRNOG00000013160	2297.39684	-1.20304	Sash1
ENSRNOG00000026607	1449.69893	-1.2030239	Tnfsf18
ENSRNOG00000004882	114.904537	-1.1963269	Capn6
ENSRNOG00000058289	126.199276	-1.1953378	AABR07027575.1
ENSRNOG00000024372	109.818282	-1.1881727	Cwf19l2
ENSRNOG00000008116	766.071491	-1.1857103	Plpp3
ENSRNOG00000006723	2200.19694	-1.1794722	Itga11
ENSRNOG00000018804	128.125404	-1.1773496	Ripor2
ENSRNOG00000055049	683.931257	-1.1766813	Aldh1a2
ENSRNOG00000005670	140.447866	-1.1743924	Art4
ENSRNOG00000025895	2560.3365	-1.1689631	Cavin2
ENSRNOG00000007202	313.063761	-1.1666517	Sema3d
ENSRNOG00000021243	114.040007	-1.1662597	Siglec1
ENSRNOG00000015658	1026.99027	-1.153369	Sorbs1

ENSRNOG00000024870	134.394344	-1.1466651	Anks1b
ENSRNOG00000003018	3394.24845	-1.143367	Olfml2b
ENSRNOG00000001642	1285.26024	-1.1405599	Tbc1d23
ENSRNOG00000012658	873.040891	-1.1334383	Pdlim3
ENSRNOG00000033581	623.838939	-1.1325762	AABR07044388.2
ENSRNOG00000020467	868.988648	-1.1299703	Nrep
ENSRNOG00000011871	105.813584	-1.1191056	AABR07066180.1
ENSRNOG00000012095	124.906934	-1.1127831	Pkia
ENSRNOG00000019577	100.611003	-1.1125766	Adamts19
ENSRNOG00000004578	1447.88237	-1.1108404	Cthrc1
ENSRNOG00000011521	114.502312	-1.1095727	Filip1
ENSRNOG00000001302	172.448146	-1.1029023	Adora2a
ENSRNOG00000004512	304.585083	-1.1026802	Apool
ENSRNOG00000013179	2824.73064	-1.1020987	Tinagl1
ENSRNOG00000008697	6877.6321	-1.1015191	Nov
ENSRNOG00000012036	241.794391	-1.0988847	Pcsk5
ENSRNOG00000057713	556.552328	-1.0947095	Cav2
ENSRNOG00000015736	488.449477	-1.0946444	Dhrs3
ENSRNOG00000036816	5420.30077	-1.0943337	Wls
ENSRNOG00000022356	365.139823	-1.0928821	Zc2hc1a
ENSRNOG00000026293	1396.09837	-1.0886758	Jun
ENSRNOG00000000572	258.050414	-1.0876486	Chst3
ENSRNOG00000006410	126.628443	-1.0832242	Akap5
ENSRNOG00000009715	2288.97231	-1.0784967	Me1
ENSRNOG00000006833	796.361098	-1.0750697	Rb1cc1
ENSRNOG00000023261	344.297675	-1.0722594	Ube2q2l
ENSRNOG00000031092	1276.44928	-1.0709594	Rock1
ENSRNOG00000018859	305.823698	-1.0646881	Pik3ip1
ENSRNOG00000013002	708.301094	-1.0609549	Gpbp1
ENSRNOG00000016378	155.601905	-1.0596197	Map3k8
ENSRNOG00000014837	1269.88062	-1.0582314	Emilin2
ENSRNOG00000021084	262.809467	-1.0560969	AABR07006310.1
ENSRNOG00000050215	264.004552	-1.0460383	AABR07044375.1
ENSRNOG00000058973	401.591025	-1.0423239	Atad2b
ENSRNOG00000010921	388.961482	-1.0418971	Taf1d
ENSRNOG00000049873	118.581897	-1.0406122	Ap1s3
ENSRNOG00000027520	6495.18103	-1.0373131	Pls3
ENSRNOG00000018382	173.386515	-1.0356152	Inpp4b
ENSRNOG00000013017	652.746593	-1.0256441	Arnt2
ENSRNOG00000031814	1266.80653	-1.0247192	Sort1
ENSRNOG00000051440	531.531045	-1.0242338	Ppp1r12b

ENSRNOG00000004540	248.277183	-1.0211206	Clec3b
ENSRNOG00000060100	137.422981	-1.0205676	Knl1
ENSRNOG00000051548	1306.65174	-1.0184655	Lmod1
ENSRNOG00000022839	134.934983	-1.0143955	Ifit3
ENSRNOG00000056246	1819.19273	-1.013948	Gls
ENSRNOG00000004160	404.550935	-1.013314	Prps2
ENSRNOG00000013090	1474.24294	-1.012398	Gadd45g
ENSRNOG00000055564	266.988728	-1.0045132	RGD1564664
ENSRNOG00000013391	877.226839	-0.9984877	Sorbs2
ENSRNOG00000013963	6663.05255	-0.9968847	Il6st
ENSRNOG00000054488	104.055641	-0.9950732	LOC500584
ENSRNOG00000009949	1709.50788	-0.9950608	Pcdh18
ENSRNOG00000000563	1058.39713	-0.9948149	Adamts14
ENSRNOG00000008079	1218.68301	-0.9937287	Ugp2
ENSRNOG00000024345	178.847403	-0.9921251	Pard3b
ENSRNOG00000004281	304.410134	-0.9901088	Cobl
ENSRNOG00000026044	148.790561	-0.9895122	Prrg1
ENSRNOG00000006726	139.145971	-0.9889287	Zfp9
ENSRNOG00000014490	134.914926	-0.9856343	Bdh2
ENSRNOG00000012733	325.843403	-0.9846821	Ankrd12
ENSRNOG00000028623	808.826842	-0.9830159	Agpat5
ENSRNOG00000025691	100.577107	-0.9812152	Pla2g7
ENSRNOG00000027811	192.410292	-0.9801475	Lilrb4
ENSRNOG00000010529	3815.73685	-0.9796695	Thbs2
ENSRNOG00000007937	388.61651	-0.9795981	Krit1
ENSRNOG00000019336	113.069603	-0.9792025	Gata3
ENSRNOG00000005464	1356.44573	-0.9781827	Lgalsl
ENSRNOG00000016848	1883.90115	-0.976416	Fzd4
ENSRNOG00000016496	1011.1071	-0.9763943	Ctsc
ENSRNOG00000010827	237.339088	-0.9732599	Ptbp2
ENSRNOG00000003261	4109.78128	-0.9715193	Usp9x
ENSRNOG00000002947	1288.63478	-0.9702331	Dpt
ENSRNOG00000016021	443.771981	-0.9686	Lims2
ENSRNOG00000014066	916.353684	-0.9665759	Jade1
ENSRNOG00000002537	222.994664	-0.9606794	Wnk3
ENSRNOG00000046527	234.860427	-0.9602084	Phf20l1
ENSRNOG00000001584	125.308544	-0.9569004	Map3k7cl
ENSRNOG00000009503	169.294208	-0.9536513	Depdc1
ENSRNOG00000053200	238.518167	-0.9503632	AABR07068161.1
ENSRNOG00000001621	1310.53742	-0.9501315	C2cd2
ENSRNOG00000001959	177.758659	-0.9497224	Mx1
ENSRNOG00000007377	1676.02476	-0.9425526	Slit3

ENSRNOG00000043304	272.377949	-0.9410707	Apcdd1
ENSRNOG00000017879	528.572581	-0.9316981	Gab1
ENSRNOG00000003357	267085.069	-0.9304719	Col3a1
ENSRNOG00000008917	743.063445	-0.9292322	Ehbp1
ENSRNOG00000013309	260.597588	-0.9247671	Pik3ap1
ENSRNOG00000010233	22436.088	-0.922448	Cald1
ENSRNOG00000006391	500.960153	-0.9206874	Smarcad1
ENSRNOG00000013426	388.451342	-0.9177441	Mrgprf
ENSRNOG00000045683	134.438313	-0.9174485	LOC102553715
ENSRNOG00000036592	135.97836	-0.9160606	Zfp518a
ENSRNOG00000012660	24877.3641	-0.9159256	Postn
ENSRNOG00000018836	11877.1807	-0.912454	RGD1559896
ENSRNOG00000010274	1170.44825	-0.9122355	Smc4
ENSRNOG00000014361	1235.40511	-0.9116221	Edn1
ENSRNOG00000000579	1803.41603	-0.9105883	Marcks
ENSRNOG00000027839	113.447321	-0.9105785	Ptk2b
ENSRNOG00000010891	267.709668	-0.9089683	Lrrcc1
ENSRNOG00000020456	3449.46056	-0.90893	Nucb2
ENSRNOG00000001369	425.037902	-0.906596	Oas1a
ENSRNOG00000030880	2314.00896	-0.9008822	Hs6st2
ENSRNOG00000019875	718.255667	-0.9001375	Matr3
ENSRNOG00000059166	109.886864	-0.8971595	Ldb3
ENSRNOG00000020479	746.651672	-0.8968457	Pik3c2a
ENSRNOG00000004268	279.639651	-0.8952242	Zfp386
ENSRNOG00000008055	138.206843	-0.8935592	Ccne2
ENSRNOG00000023579	431.056076	-0.8926413	Tet2
ENSRNOG00000007315	519.733238	-0.8921104	Thoc2
ENSRNOG00000009954	1454.17535	-0.8904286	Cacul1
ENSRNOG00000012060	902.00325	-0.8902568	Gucy1b3
ENSRNOG00000008626	690.404757	-0.8896554	Manea
ENSRNOG00000033119	1010.74841	-0.8876061	Plcb4
ENSRNOG00000019108	685.336058	-0.8861118	Rmi1
ENSRNOG00000032150	113.71532	-0.8845775	Adcy2
ENSRNOG00000009951	660.279132	-0.880538	Aifl1
ENSRNOG00000021776	218.316139	-0.8781248	Cenpc
ENSRNOG00000049097	467.002319	-0.877139	Rpl7a
ENSRNOG00000000657	824.500806	-0.8771135	Nek7
ENSRNOG00000009208	135.664477	-0.8765401	Pibf1
ENSRNOG00000011668	374.301009	-0.8753699	Nfil3
ENSRNOG00000054011	471.881401	-0.8750767	Tbc1d8b
ENSRNOG00000013121	337.52289	-0.8717489	Mier3
ENSRNOG00000036604	981.220683	-0.8711109	Ifit2
ENSRNOG00000006227	509.632646	-0.8708449	Ifih1

ENSRNOG00000003674	442.55051	-0.8696573	Pir
ENSRNOG00000001030	4900.40707	-0.8689396	Tsc22d1
ENSRNOG00000014275	285.397076	-0.8686451	AABR07026291.1
ENSRNOG00000002886	20842.5329	-0.868593	Myh10
ENSRNOG00000025155	1114.4411	-0.8684199	Lmtk2
ENSRNOG00000004696	1180.82811	-0.8658757	Arhgap5
ENSRNOG00000033220	407.091986	-0.86478	Oas1b
ENSRNOG00000055567	342.667056	-0.8645925	Fmnl2
ENSRNOG00000018102	777.827186	-0.8644917	Coa5
ENSRNOG00000007124	561.318795	-0.8637535	Krcc1
ENSRNOG00000005814	140.116792	-0.8628186	Katnbl1
ENSRNOG00000004908	821.879789	-0.8624599	Smc6
ENSRNOG00000022871	217.654519	-0.861522	LOC691170
ENSRNOG00000004198	252.449456	-0.8586605	Stxbp6
ENSRNOG00000014928	130.701156	-0.8579091	Apba1
ENSRNOG00000014501	575.804848	-0.8567097	Zfp638
ENSRNOG00000008788	385.576529	-0.8561158	Mpp5
ENSRNOG00000002280	921.377676	-0.8540381	Sh3bgrl
ENSRNOG00000014867	216.313901	-0.8537019	Synpo2
ENSRNOG00000013306	276.56404	-0.8532225	Pcdh20
ENSRNOG00000005917	1258.76438	-0.8520172	Pawr
ENSRNOG00000032401	122.835533	-0.8507359	H3f3c
ENSRNOG00000002751	118.532536	-0.8498363	Zdhhc15
ENSRNOG00000018951	1427.4829	-0.84836	Col4a5
ENSRNOG00000015552	479.960105	-0.8459282	Ppil4
ENSRNOG00000022218	576.47406	-0.8458874	Ifi44
ENSRNOG00000028585	1601.8485	-0.8452242	Tceal8
ENSRNOG00000013321	588.099643	-0.8448117	Dock11
ENSRNOG00000009656	112.160611	-0.8432701	Rspo1
ENSRNOG00000006487	363.925235	-0.8431414	Casp8ap2
ENSRNOG00000010977	1419.29499	-0.8427414	Igfbp6
ENSRNOG00000011606	116.750931	-0.8420109	AABR07061825.1
ENSRNOG00000028814	802.961714	-0.8411235	Oasl2
ENSRNOG00000016752	554.688885	-0.8409758	Crispld2
ENSRNOG00000004610	2773.00978	-0.8402494	Lum
ENSRNOG00000032258	189.004059	-0.8402041	Swt1
ENSRNOG00000009104	304.940823	-0.8401225	AABR07029605.1
ENSRNOG00000004496	4082.78338	-0.8383803	Rock2
ENSRNOG00000007821	765.855662	-0.8381305	Dyrk2
ENSRNOG00000000082	273.82586	-0.8355646	Hltf
ENSRNOG00000004679	103.24842	-0.833271	Fign

ENSRNOG00000015334	314.001795	-0.8324163	Fcho2
ENSRNOG00000010832	569.129782	-0.832054	Pdgfrl
ENSRNOG00000059016	754.081457	-0.8320511	Tspan12
ENSRNOG00000008659	3489.58102	-0.8302486	Arhgap21
ENSRNOG00000006384	484.113011	-0.8300103	Ddx58
ENSRNOG00000054901	299.085128	-0.8298292	Rif1
ENSRNOG00000003242	224.661184	-0.8291465	Gulp1
ENSRNOG00000016243	148.577727	-0.8255637	Casq2
ENSRNOG00000008859	250.498456	-0.8245133	Tank
ENSRNOG00000002730	438.332472	-0.8241989	Rgs5
ENSRNOG00000010840	1262.40285	-0.8228473	Adamtsl3
ENSRNOG00000003439	254.58109	-0.8226815	Il15
ENSRNOG00000005907	210.184312	-0.8223509	Rad18
ENSRNOG00000005158	101.700783	-0.8220447	Slc24a5
ENSRNOG00000008941	3867.20263	-0.8219694	Ets1
ENSRNOG00000012414	721.012882	-0.8207207	Rhobtb3
ENSRNOG00000026186	588.945187	-0.820518	Syde2
ENSRNOG00000018517	302.392063	-0.8197584	Trim21
ENSRNOG00000004677	2474.50532	-0.8180207	Zeb2
ENSRNOG00000011720	152.620483	-0.8163119	L3mbtl3
ENSRNOG00000021206	115.186107	-0.8143282	Pla2g16
ENSRNOG00000012067	1572.24535	-0.8139306	Fam111a
ENSRNOG00000006789	432.921209	-0.8134037	Ddit3
ENSRNOG00000018247	366.05414	-0.8127638	Dhx58
ENSRNOG00000011619	557.091353	-0.8114716	Myo9a
ENSRNOG00000003350	333.973976	-0.8072116	Mospd2
ENSRNOG00000022141	443.527344	-0.8056446	Ctdspl2
ENSRNOG00000009694	760.709343	-0.8048118	Bmp4
ENSRNOG00000024705	638.560415	-0.8041047	Rarres2
ENSRNOG00000028895	287.501844	-0.8041013	Rtp4
ENSRNOG00000008934	192.739113	-0.8032476	Tmem65
ENSRNOG00000007197	180.452473	-0.8031279	Nr1h4
ENSRNOG00000039091	469.385021	-0.8026195	Pnpla8
ENSRNOG00000013574	485.827482	-0.8010335	Appl1
ENSRNOG00000056735	175.56954	-0.7992857	Zc4h2
ENSRNOG00000015986	4868.12433	-0.7987804	Rassf8
ENSRNOG00000036913	570.462756	-0.7982717	RGD1309621
ENSRNOG00000010107	2207.96715	-0.7980679	AABR07025295.1
ENSRNOG00000037198	484.079868	-0.7946383	Usp18
ENSRNOG00000014031	173.54895	-0.7932565	Esco1
ENSRNOG00000005391	193.848414	-0.7925756	Prex2
ENSRNOG00000052775	202.51426	-0.7898744	RGD1305938

ENSRNOG00000017684	172.466543	-0.7897403	Fbxl22
ENSRNOG00000018937	781.626763	-0.7887653	Gstm2
ENSRNOG00000010077	1117.74486	-0.7885576	Smarcd3
ENSRNOG00000047133	136.476157	-0.7882552	Jrkl
ENSRNOG00000014791	1356.81535	-0.78779	Peg3
ENSRNOG00000006632	1053.84534	-0.7876067	Rps6ka3
ENSRNOG00000052247	387.318904	-0.7852589	Manba
ENSRNOG00000005854	139.774905	-0.7838483	Angpt1
ENSRNOG00000001825	105.012696	-0.7838218	AABR07034767.1
ENSRNOG00000011016	776.131501	-0.7835963	Slc7a2
ENSRNOG00000008314	268.049581	-0.7835144	Orc3
ENSRNOG00000039183	168.604612	-0.7826788	Cip2a
ENSRNOG00000059243	142.613124	-0.7818899	AABR07051787.1
ENSRNOG00000013917	1255.32181	-0.7815848	Igsf10
ENSRNOG00000016208	188.276251	-0.7808554	Setbp1
ENSRNOG00000055225	122.022763	-0.7795912	RF02266
ENSRNOG00000031801	886.852228	-0.7782433	Ephb3
ENSRNOG00000006324	438.250492	-0.7782317	Trpc6
ENSRNOG00000006749	491.57185	-0.7779508	Tmtc3
ENSRNOG00000022769	322.437281	-0.7778367	Sp100
ENSRNOG00000015577	165.656032	-0.7766124	Lpar6
ENSRNOG00000025042	568.174388	-0.7765338	Pde3a
ENSRNOG00000011586	558.648172	-0.7739694	LOC100909712
ENSRNOG00000011859	437.698722	-0.7731094	Eif5a2
ENSRNOG00000010744	2223.1089	-0.7723509	Nrp1
ENSRNOG00000008445	974.628127	-0.7718003	Dact1
ENSRNOG00000025539	175.859024	-0.7714945	Vps13a
ENSRNOG00000009338	332.801429	-0.770692	Kras
ENSRNOG00000043387	2232.65972	-0.7696349	Cpe
ENSRNOG00000008039	528.932547	-0.7682223	Cul5
ENSRNOG00000004033	567.662988	-0.768166	Sema6a
ENSRNOG00000010966	57097.5269	-0.7678147	Itgb1
ENSRNOG00000004304	612.089257	-0.7669548	Syap1
ENSRNOG00000003825	987.469265	-0.7664697	Wdr75
ENSRNOG00000017980	128.854483	-0.7664423	Itgal
ENSRNOG00000053196	1038.89447	-0.7659827	Slc48a1
ENSRNOG00000054669	273.598565	-0.7657961	RGD1305110
ENSRNOG00000009311	481.515238	-0.7641022	Fstl3
ENSRNOG00000009990	962.475934	-0.7627577	Zranb2
ENSRNOG00000005730	279.626774	-0.7626016	Pcmtd1
ENSRNOG00000001317	336.404094	-0.7624005	Zfp68
ENSRNOG00000015691	129.045259	-0.7623875	Fam212b

ENSRNOG00000008151	468.591857	-0.7615193	Plscr4
ENSRNOG00000020024	272.787964	-0.7609964	Taf7
ENSRNOG00000007662	202.214112	-0.760145	Zfp800
ENSRNOG00000057464	734.543572	-0.7599841	Fmr1
ENSRNOG00000005070	226.356766	-0.7590172	Spopl
ENSRNOG00000015255	166.13794	-0.757992	Haus3
ENSRNOG00000011951	4710.87162	-0.7571883	Plk2
ENSRNOG00000020904	469.990459	-0.7569929	Cdc42ep2
ENSRNOG00000007682	147.443881	-0.7554869	Gria3
ENSRNOG00000014426	11773.396	-0.7545532	Lox
ENSRNOG00000011137	181.797533	-0.7542148	Zbtb41
ENSRNOG00000045740	613.420868	-0.7528506	Tmx3
ENSRNOG00000014673	151.441464	-0.7524992	Eri2
ENSRNOG00000006432	356.055405	-0.7524738	Trnt1
ENSRNOG00000009980	2375.78066	-0.7503674	Plpp1
ENSRNOG00000021242	348.640813	0.75017846	Adam33
ENSRNOG00000032238	1571.1491	0.75211836	Plcd1
ENSRNOG00000020481	339.480999	0.75299756	Pafah1b3
ENSRNOG00000031420	928.444094	0.75413229	Hyal2
ENSRNOG00000025619	202.912696	0.75521995	Ap1g2
ENSRNOG00000020657	4790.37554	0.75711118	Shc1
ENSRNOG00000016560	603.585129	0.76038084	Card19
ENSRNOG00000017328	155.137756	0.76072926	Pter
ENSRNOG00000007818	595.144625	0.76159946	Slc45a4
ENSRNOG00000008243	375.827231	0.76166306	Slc43a1
ENSRNOG00000015439	4996.56861	0.76223908	Man2a1
ENSRNOG00000007412	281.860881	0.76302153	Dok1
ENSRNOG00000000177	419.130713	0.76403532	Plpp2
ENSRNOG00000015118	192.090444	0.76421553	Cpped1
ENSRNOG00000013946	830.867148	0.76465821	Rnf149
ENSRNOG00000004903	455.417189	0.76503708	Ebp
ENSRNOG00000014504	5922.38445	0.765143	Il1r1
ENSRNOG00000004258	234.527887	0.76649757	Chst7
ENSRNOG00000054549	2206.81384	0.76679148	Lss
ENSRNOG00000019388	544.645153	0.76778518	Egfl7
ENSRNOG00000027880	159.847133	0.76822507	Nbeal2
ENSRNOG00000018659	3251.31829	0.76893	Csf1
ENSRNOG00000007230	165.948662	0.76897088	Kank3
ENSRNOG00000052157	354.214623	0.77106499	Nav3
ENSRNOG00000002969	582.828583	0.77242104	Itpkb
ENSRNOG00000007993	267.263479	0.77251326	Sh3tc1
ENSRNOG00000017463	250.35015	0.77332913	Bloc1s3
ENSRNOG00000031743	411.265165	0.77451136	Gbp2

ENSRNOG00000018785	129.071124	0.77517045	Slc16a13
ENSRNOG00000020238	437.164351	0.77532051	Plekhh3
ENSRNOG00000024566	317.212904	0.77551589	Sh3d21
ENSRNOG00000046560	752.396511	0.77696024	AC109096.1
ENSRNOG00000009037	4676.20477	0.77736958	Sulf1
ENSRNOG00000013604	2383.07658	0.77982585	Gpx4
ENSRNOG00000029333	177.893633	0.78025159	Echdc2
ENSRNOG00000057619	159.492175	0.78035408	LOC100912068
ENSRNOG00000051039	232.926117	0.78232886	LOC108349594
ENSRNOG00000043102	469.744751	0.7833876	Bahcc1
ENSRNOG00000008012	276.746057	0.78718136	Abcb1b
ENSRNOG00000049937	225.385037	0.78934598	Pdxk
ENSRNOG00000017839	795.555642	0.79317838	Ercc1
ENSRNOG00000021185	433.403221	0.79371389	Bola1
ENSRNOG00000018019	165.019854	0.79487341	Hspa12a
ENSRNOG00000026519	1846.6958	0.79523624	LOC100364062
ENSRNOG00000018237	5080.22184	0.79757001	Gstp1
ENSRNOG00000013783	3207.44028	0.79803232	Efhd2
ENSRNOG00000002322	211.049889	0.79866851	RGD1310587
ENSRNOG00000048915	355.007771	0.79899136	Twf2
ENSRNOG00000018778	566.381388	0.79981649	Cadm1
ENSRNOG00000026435	133.668528	0.80070875	Arid3a
ENSRNOG00000010882	1120.6685	0.80184039	Sptlc1
ENSRNOG00000039745	101.872526	0.80387804	Pm20d1
ENSRNOG00000001928	479.85038	0.80406336	Il1rap
ENSRNOG00000032788	990.177545	0.80415096	Dysf
ENSRNOG00000018874	331.582659	0.80487929	Phf19
ENSRNOG00000001729	611.489093	0.80517346	Xxylt1
ENSRNOG00000022067	258.653602	0.80554131	Tlr5
ENSRNOG00000021812	150.583616	0.80572333	Scx
ENSRNOG00000010058	2665.03377	0.805752	Spry2
ENSRNOG00000053498	331.752134	0.80720908	Dnajc22
ENSRNOG00000050478	150.343553	0.8085257	LOC102549173
ENSRNOG00000009068	5446.49349	0.80871404	Phlda3
ENSRNOG00000024410	433.808702	0.80951227	Blvrb
ENSRNOG00000020088	359.229969	0.81169831	Klhl26
ENSRNOG00000024349	182.642583	0.81220971	Cbarp
ENSRNOG00000053272	350.360649	0.81411238	Chi3l1
ENSRNOG00000029141	258.623696	0.81440123	Trabd2b
ENSRNOG00000001229	5784.40579	0.81442017	Col18a1
ENSRNOG00000000967	2045.69856	0.82327338	Aacs
ENSRNOG00000004863	129.618347	0.82345979	Mpped2
ENSRNOG00000003144	334.814645	0.82393595	Gprc5c

ENSRNOG00000011329	23223.3526	0.82600697	Pkm
ENSRNOG00000016544	153.70181	0.8271962	Arhgef28
ENSRNOG00000019211	4317.69437	0.8280653	Olfml3
ENSRNOG00000011967	250.841126	0.83168449	Nphp4
ENSRNOG00000005053	168.107048	0.83543919	Egln3
ENSRNOG00000012772	843.608223	0.8379278	Nqo1
ENSRNOG00000013659	925.117383	0.83868231	Tex2
ENSRNOG00000059956	239.414868	0.84117437	Bcl6b
ENSRNOG00000014963	184.714576	0.84378974	Adgrg1
ENSRNOG00000015113	117.429929	0.84574371	Mocos
ENSRNOG00000004110	1342.66991	0.84680848	Trib2
ENSRNOG00000023348	165.664513	0.84709223	Tbc1d2
ENSRNOG00000055527	198.025068	0.84800884	Arap3
ENSRNOG00000005935	1347.44468	0.84802618	A3galt2
ENSRNOG00000033235	561.003965	0.84853588	Relb
ENSRNOG00000025448	1268.58686	0.84986576	Limd2
ENSRNOG00000004367	1911.19404	0.85023426	Elk3
ENSRNOG00000020726	1818.49137	0.85058332	Sipa1
ENSRNOG00000025143	100.319814	0.85144081	Icam2
ENSRNOG00000005393	393.484158	0.85278159	Sertad2
ENSRNOG00000029614	846.787534	0.85352261	Robo1
ENSRNOG00000017212	301.579434	0.85402877	Spsb1
ENSRNOG00000008936	795.826634	0.85470771	Map3k6
ENSRNOG00000049965	218.826683	0.85494864	LOC103693608
ENSRNOG00000001214	3939.12649	0.85691798	Pfkl
ENSRNOG00000018669	328.977478	0.85840131	Jak3
ENSRNOG00000009419	1569.59405	0.85849257	Ptprg
ENSRNOG00000028801	282.80868	0.85899923	Gsap
ENSRNOG00000013668	2943.88063	0.86058569	Capg
ENSRNOG00000045558	1038.35301	0.86454903	Cd34
ENSRNOG00000010629	768.170939	0.86519503	Nod1
ENSRNOG00000050108	192.23202	0.86535641	LOC100911319
ENSRNOG00000007827	250.843535	0.86837581	Cox4i2
ENSRNOG00000021174	112.341483	0.86885152	Macrod1
ENSRNOG00000019698	1336.45752	0.86951535	Ssbp4
ENSRNOG00000010165	5349.58122	0.86997612	Tnfaip2
ENSRNOG00000008048	704.534984	0.87049062	Plscr1
ENSRNOG00000013552	3909.61764	0.87176378	Scd
ENSRNOG00000015618	268.136683	0.87453761	Wnt5a
ENSRNOG00000010213	209.361536	0.8774042	Fgd5
ENSRNOG00000036693	584.779255	0.88083555	Slc25a10
ENSRNOG00000012108	126.474018	0.88146173	Thsd1
ENSRNOG00000018397	139.05283	0.88461296	Dnph1

ENSRNOG00000017610	498.365095	0.88747092	Nedd4l
ENSRNOG00000013069	552.515585	0.88801831	Sapcd2
ENSRNOG00000010580	1477.21725	0.88884334	Acot7
ENSRNOG00000012939	306.364558	0.88906127	Abca7
ENSRNOG00000004273	3904.95772	0.89335575	Ifitm1
ENSRNOG00000010170	8474.52705	0.89489131	Tubb4b
ENSRNOG00000000503	1101.10492	0.89756731	Ppard
ENSRNOG00000028415	2456.24825	0.89811548	Cdc20
ENSRNOG00000029768	325.451396	0.89989376	Ccl12
ENSRNOG00000016846	742.268503	0.90039182	Pik3cd
ENSRNOG00000013720	8193.40487	0.90112373	Aebp1
ENSRNOG00000021104	5655.74488	0.90129026	Emp3
ENSRNOG00000007027	148.582073	0.90163248	Hgf
ENSRNOG00000007582	948.693219	0.90177709	Zswim4
ENSRNOG00000019265	210.533321	0.90294197	Pcdh12
ENSRNOG00000020173	1140.58418	0.90494102	Tie1
ENSRNOG00000017146	664.383744	0.90551523	Nfatc1
ENSRNOG00000061876	1528.27147	0.90616012	Tas1r2
ENSRNOG00000016968	1431.10279	0.90630609	Gramd4
ENSRNOG00000036677	593.239126	0.90739152	Slc16a3
ENSRNOG00000005731	146.157317	0.90896932	Birc3
ENSRNOG00000000787	178.579309	0.91056811	AABR07044364.1
ENSRNOG00000018384	4581.90846	0.91136358	Adam12
ENSRNOG00000009712	1375.19075	0.91195356	Gale
ENSRNOG00000029330	115.278481	0.91356071	Ca5b
ENSRNOG00000020533	22100.9232	0.91415449	Htra1
ENSRNOG00000012216	334.979316	0.91576886	Tgfbi
ENSRNOG00000004888	445.439544	0.91581336	Spred2
ENSRNOG00000015495	235.512337	0.91630399	Slc25a37
ENSRNOG00000028569	121.368013	0.91802549	Arhgap27
ENSRNOG00000017197	297.289469	0.91807204	Pdgfb
ENSRNOG00000025527	637.817407	0.92130077	Mtcl1
ENSRNOG00000007060	2975.53142	0.92282671	Plin2
ENSRNOG00000024818	3772.37713	0.92380802	Eva1b
ENSRNOG00000036649	390.388031	0.92486153	Pwwp2b
ENSRNOG00000021438	1123.46645	0.92593909	Tuba1c
ENSRNOG00000052296	409.018578	0.93333146	Shank3
ENSRNOG00000011781	825.033217	0.93422682	Oplah
ENSRNOG00000049437	1542.40963	0.93474114	Gpc1
ENSRNOG00000024703	740.665899	0.93507774	Dock5
ENSRNOG00000014243	2049.14084	0.93546014	Pear1
ENSRNOG00000003650	140.127043	0.93713577	Nt5c

ENSRNOG00000011321	757.648422	0.93757591	Rftn1
ENSRNOG00000045779	290.595957	0.93854831	AABR07039210.2
ENSRNOG00000008401	191.51418	0.93872898	Card10
ENSRNOG00000013581	1158.29949	0.94111373	Extl3
ENSRNOG00000000151	675.793149	0.941919	Ldlrap1
ENSRNOG00000002810	1720.41968	0.94329532	Gfpt2
ENSRNOG00000057153	573.972244	0.94347957	Pla1a
ENSRNOG00000048932	160.815108	0.94405407	Smagp
ENSRNOG00000059015	1516.46283	0.9451493	Triobp
ENSRNOG00000011969	468.254314	0.94532425	Dock9
ENSRNOG00000009850	1079.99534	0.9458898	St3gal4
ENSRNOG00000019048	4236.29609	0.94825387	Sod2
ENSRNOG00000004049	401.720496	0.94927119	Baiap2
ENSRNOG00000007946	1924.99573	0.95147537	Bcl2l1
ENSRNOG00000056585	4240.50262	0.9537615	Fscn1
ENSRNOG00000018992	8555.9044	0.95451951	Dpysl3
ENSRNOG00000012952	698.734873	0.957657	Lrig1
ENSRNOG00000020401	455.985602	0.95939068	Adcy4
ENSRNOG00000012950	142.312523	0.963241	Efr3b
ENSRNOG00000018964	1168.60025	0.964406	Gss
ENSRNOG00000009870	141.084579	0.9657103	Tmem88
ENSRNOG00000019741	830.693193	0.96581153	Isyna1
ENSRNOG00000023007	187.701494	0.96603431	Col6a6
ENSRNOG00000003635	425.501622	0.96715835	Disp1
ENSRNOG00000015583	481.461622	0.97022931	Dnal4
ENSRNOG00000021102	172.239956	0.97060749	Scn1b
ENSRNOG00000021447	210.329914	0.97077777	Prr7
ENSRNOG00000022309	1039.51664	0.97109576	Frem1
ENSRNOG00000061519	528.085293	0.97170076	Asap2
ENSRNOG00000049361	943.56753	0.97183875	Gas7
ENSRNOG00000000502	205.06332	0.97202191	Def6
ENSRNOG00000019689	222.029864	0.97394568	Vwf
ENSRNOG00000013324	1452.02889	0.97457442	Cdh5
ENSRNOG00000002511	167.246745	0.97616097	Flt4
ENSRNOG00000011411	242.793835	0.97865269	Adgrg6
ENSRNOG00000013376	1634.00018	0.98119052	Mvd
ENSRNOG00000009370	188.769183	0.98379261	Tbkbp1
ENSRNOG00000028036	2644.01645	0.98404817	Adamts7
ENSRNOG00000016701	242.723919	0.98530558	Gng8
ENSRNOG00000015971	1073.54056	0.98684017	Slc12a2
ENSRNOG00000008215	2141.26574	0.99208496	Trim47
ENSRNOG00000045999	111.310534	0.99363344	Tnfaip8l1

ENSRNOG00000058364	113.929643	0.99376068	AABR07051515.1
ENSRNOG00000007541	2085.5941	0.99623591	Fhl3
ENSRNOG00000049056	142.976436	0.99704118	AABR07051450.1
ENSRNOG00000013196	109.367349	1.00591112	Dok5
ENSRNOG00000019403	603.307675	1.00933305	Afap1l1
ENSRNOG00000060381	1480.79187	1.01282147	Col15a1
ENSRNOG00000020277	269.257801	1.01352855	Cntnap1
ENSRNOG00000007324	1153.45207	1.01691461	Plxna2
ENSRNOG00000021125	3683.97472	1.01710945	Prdx5
ENSRNOG00000052795	1199.58806	1.02112785	Itpr3
ENSRNOG00000017489	266.929817	1.02117972	Gse1
ENSRNOG00000024688	522.887905	1.02213119	Erfe
ENSRNOG00000006615	2149.4677	1.02384637	Mtap
ENSRNOG00000003064	375.037551	1.02523478	Bst1
ENSRNOG00000004019	987.3165	1.03381529	Phlda1
ENSRNOG00000013545	1920.27055	1.03416596	Polr2e
ENSRNOG00000015967	1887.31794	1.03719572	Sh3bgrl3
ENSRNOG00000015455	478.52653	1.03741562	Spr
ENSRNOG00000054374	166.523663	1.0385939	Smim29
ENSRNOG00000013463	238.862389	1.04125348	Kcnj8
ENSRNOG00000020703	1007.90344	1.04183479	Sipa1l3
ENSRNOG00000047924	156.674945	1.04536806	Zbtb7c
ENSRNOG00000009113	926.292795	1.04664097	Marcksl1
ENSRNOG00000037909	2212.96518	1.04690329	Ppm1f
ENSRNOG00000016257	4313.49527	1.04735209	Cotl1
ENSRNOG00000009862	240.627517	1.04759683	Olfm1
ENSRNOG00000003088	928.76117	1.04936176	Arhgap31
ENSRNOG00000024846	2536.58875	1.04941318	Ier5l
ENSRNOG00000002093	1037.87489	1.05116445	Tgfbr3
ENSRNOG00000005861	2290.19602	1.05270312	Hsd11b1
ENSRNOG00000009348	556.258926	1.05402489	Nos3
ENSRNOG00000006867	147.057872	1.05833122	Etv1
ENSRNOG00000007567	929.361394	1.0591489	Rem1
ENSRNOG00000004823	287.183281	1.06001631	Dock4
ENSRNOG00000017434	311.604777	1.06038747	Mgat3
ENSRNOG00000010111	154.782491	1.06211175	Exoc3l4
ENSRNOG00000008425	2320.86227	1.06313686	Nav1
ENSRNOG00000006548	5345.93338	1.06358435	Mrc2
ENSRNOG00000037113	1500.14585	1.069409	Slfn2
ENSRNOG00000014648	1305.9005	1.0713877	Efnb2
ENSRNOG00000004482	126.552003	1.07232415	Ccdc88c
ENSRNOG00000018452	186.820024	1.08348434	Unc13a

ENSRNOG00000028650	2127.2776	1.0893206	Inf2
ENSRNOG00000021916	121.888074	1.09014075	Slc16a12
ENSRNOG00000006033	358.873982	1.0908557	Spon2
ENSRNOG00000017208	3200.209	1.09712498	Cspg4
ENSRNOG00000014357	330.726125	1.09750296	Gja4
ENSRNOG00000002926	2494.63685	1.10006778	Uap1
ENSRNOG00000005024	528.460498	1.10189369	Cdkn2aipnl
ENSRNOG00000020573	172.095509	1.10473162	Efna1
ENSRNOG00000003069	1313.63684	1.10829703	Cd38
ENSRNOG00000001442	2793.93619	1.10996435	Por
ENSRNOG00000004560	223.784787	1.11045678	Cacna1b
ENSRNOG00000008133	104.27044	1.11696362	Mfng
ENSRNOG00000007254	249.882349	1.12003724	Ttc9
ENSRNOG00000001728	250.034061	1.12105144	Fam43a
ENSRNOG00000003120	5153.20844	1.12325494	Prelp
ENSRNOG00000001785	1184.81297	1.1260391	Etv5
ENSRNOG00000021441	413.494331	1.12840065	Reln
ENSRNOG00000037931	737.920371	1.1410126	Plaur
ENSRNOG00000000187	328.304249	1.14453112	Csf2rb
ENSRNOG00000049471	396.623341	1.1456627	Steap3
ENSRNOG00000007839	154.145633	1.15381827	Slc16a7
ENSRNOG00000016326	772.945559	1.16050033	Cx3cl1
ENSRNOG00000038784	490.874624	1.16126031	Piezo2
ENSRNOG00000022392	481.553149	1.16455011	Hspb8
ENSRNOG00000011775	110.4816	1.16517919	Mfap3l
ENSRNOG00000014197	355.178874	1.16689108	Tmem51
ENSRNOG00000026902	100.198782	1.16805301	Lyve1
ENSRNOG00000004757	298.453073	1.17367865	Tmem158
ENSRNOG00000024243	591.351611	1.1810291	Cadm4
ENSRNOG00000021062	1197.04894	1.1823727	Fxyd5
ENSRNOG00000021004	214.281248	1.18274532	Rasip1
ENSRNOG00000059344	2093.14252	1.18299796	Tpcn1
ENSRNOG00000026053	435.197215	1.18304265	Grem1
ENSRNOG00000048291	878.755626	1.18413154	Tbcc
ENSRNOG00000002771	111.820403	1.18578444	Ereg
ENSRNOG00000020193	224.614646	1.18717721	Runx2
ENSRNOG00000010417	170.650296	1.18920549	Nol4l
ENSRNOG00000014658	167.358286	1.19431204	Zfp423
ENSRNOG00000013064	775.598536	1.19572117	Lrp8
ENSRNOG00000030404	381.664202	1.19882629	Arrb1
ENSRNOG00000021244	716.741201	1.20065564	Hspa12b
ENSRNOG00000042905	592.9691	1.20271306	RT1-T24-4
ENSRNOG00000007687	317.211842	1.21044855	Sema7a

ENSRNOG00000019752	1040.60319	1.22008129	Slc29a1
ENSRNOG00000013927	119.597339	1.22047251	Jag2
ENSRNOG00000005287	437.455777	1.22544952	Syne3
ENSRNOG00000000569	293.767191	1.22565809	Vsir
ENSRNOG00000001314	3870.8876	1.22640945	Fam20c
ENSRNOG00000010753	813.809271	1.22773636	Aig1
ENSRNOG00000008099	231.445321	1.23041261	Galnt12
ENSRNOG00000008885	1039.11695	1.2414205	Chst11
ENSRNOG00000016248	331.769959	1.24491634	Sox18
ENSRNOG00000016980	136.170581	1.24639883	Qprt
ENSRNOG00000051564	898.708146	1.24717054	Rap2a
ENSRNOG00000011914	548.450119	1.249046	Dtnb
ENSRNOG00000006877	2034.06373	1.24990243	Efnb1
ENSRNOG00000011101	609.663435	1.25667511	Twist1
ENSRNOG00000009389	905.292429	1.25798584	Ripk2
ENSRNOG00000017149	1084.72941	1.25893719	Fam131b
ENSRNOG00000023536	398.448505	1.26888785	Adgrd1
ENSRNOG00000004417	184.510778	1.26971245	Fam117a
ENSRNOG00000028198	3003.05711	1.27188393	Sh2b3
ENSRNOG00000019536	732.255778	1.28022667	Smim3
ENSRNOG00000047300	138.63013	1.28333783	Bdkrb2
ENSRNOG00000011921	971.447197	1.29184218	Dusp4
ENSRNOG00000009369	844.23219	1.29792398	Tor4a
ENSRNOG00000060410	444.070095	1.30095913	Pcdh1
ENSRNOG00000010718	1019.08587	1.32060194	Gpr153
ENSRNOG00000055751	464.623429	1.3216973	P3h2
ENSRNOG00000006604	1721.86685	1.32423204	Thy1
ENSRNOG00000042411	162.236633	1.32463733	Rps6ka1
ENSRNOG00000009222	739.616639	1.32977035	Epha2
ENSRNOG00000031232	3452.37467	1.33255028	Nrp2
ENSRNOG00000000442	329.223624	1.33539235	Notch4
ENSRNOG00000055305	1244.46838	1.33674128	Parvb
ENSRNOG00000046848	251.751104	1.34230321	PCOLCE2
ENSRNOG00000012830	356.980505	1.35109047	Paqr8
ENSRNOG00000015441	1809.4798	1.3577386	Il4r
ENSRNOG00000014776	988.356677	1.36728053	Adcy7
ENSRNOG00000003866	314.905489	1.37781037	Cxcr4
ENSRNOG00000013791	2832.42819	1.37930069	Enpp3
ENSRNOG00000003732	1551.0737	1.38154631	Flrt2
ENSRNOG00000012049	154.997653	1.38742022	Sox7
ENSRNOG00000033090	3743.74435	1.38900833	Ltbp1
ENSRNOG00000012782	2427.73941	1.39223058	Tmem2
ENSRNOG00000001065	2212.37944	1.40023516	Cyth3

ENSRNOG00000034177	128.882767	1.40187798	Efna5
ENSRNOG00000011228	176.36456	1.40405196	Layn
ENSRNOG00000051915	313.155272	1.40759928	Spred3
ENSRNOG00000033835	765.346841	1.41180369	Dnm1
ENSRNOG00000010635	2111.12933	1.41310321	Igfbp4
ENSRNOG00000026136	179.330668	1.41744433	Tnfaip8
ENSRNOG00000007118	108.957346	1.41886877	Eva1a
ENSRNOG00000021750	1075.71312	1.41912796	Id1
ENSRNOG00000024594	163.336297	1.42363537	Fhdc1
ENSRNOG00000000805	966.647574	1.43272097	Gja1
ENSRNOG00000014987	349.529696	1.43290746	Mdfi
ENSRNOG00000016640	145.244255	1.4362042	Dner
ENSRNOG00000014232	220.064156	1.44858114	P2ry1
ENSRNOG00000000017	609.503154	1.4531388	Steap1
ENSRNOG00000002667	158.014487	1.45636899	Lamc2
ENSRNOG00000012881	683.063704	1.46093582	Fgl2
ENSRNOG00000026647	1857.12599	1.4619828	Cxcl16
ENSRNOG00000057347	982.445195	1.46468534	Cebpb
ENSRNOG00000052564	9197.14097	1.46739265	Gpx3
ENSRNOG00000002802	532.990457	1.46800801	Cxcl1
ENSRNOG00000014011	159.45093	1.48132492	Dll4
ENSRNOG00000046829	254.990856	1.48318449	Kdr
ENSRNOG00000018752	172.982438	1.48529095	Clcf1
ENSRNOG00000003947	900.545773	1.48584509	Ntn1
ENSRNOG00000014879	1906.90948	1.48592868	Ttc7a
ENSRNOG00000037275	204.625791	1.49617608	Tlcd2
ENSRNOG00000052391	2044.18608	1.51490375	Sh3bp5
ENSRNOG00000020030	317.805217	1.54520931	Crlf1
ENSRNOG00000009354	117.262956	1.54607092	Nrarp
ENSRNOG00000016182	307.167846	1.55820429	Tgfa
ENSRNOG00000017676	300.370375	1.55992227	Plvap
ENSRNOG00000006076	1156.59036	1.58313478	Steap2
ENSRNOG00000028043	147.710983	1.58848566	Cxcl3
ENSRNOG00000009803	121.869346	1.59118617	LOC100362176
ENSRNOG00000058111	171.5167	1.59759084	Itga2
ENSRNOG00000016013	1298.99838	1.63130589	Gprc5b
ENSRNOG00000002879	2050.71516	1.65407566	Psen2
ENSRNOG00000008492	413.411297	1.65806081	Cfap45
ENSRNOG00000019549	10765.155	1.67384618	Akap12
ENSRNOG00000039668	6022.78839	1.69353433	Col8a1
ENSRNOG00000047734	928.819044	1.69535326	Chst2
ENSRNOG00000018500	1824.9915	1.69809152	AABR07028615.1

ENSRNOG00000013994	2341.97851	1.7184423	Enpp1
ENSRNOG00000054957	110.369558	1.76607114	Sfrp4
ENSRNOG00000000640	577.816068	1.77375277	Egr2
ENSRNOG00000011680	153.273047	1.77793838	Il16
ENSRNOG00000020546	193.339392	1.79161325	Lipe
ENSRNOG00000014424	855.121549	1.79648631	RGD1563354
ENSRNOG00000011648	3429.95127	1.82244671	Aqp1
ENSRNOG00000059765	167.241576	1.82773395	Asic1
ENSRNOG00000052880	192.930584	1.83196855	Prph
ENSRNOG00000051372	250.473345	1.83228402	Mycn
ENSRNOG00000018797	141.444749	1.83569531	Myrip
ENSRNOG00000010076	1381.34493	1.84685708	Pkp1
ENSRNOG00000002930	348.163218	1.86637808	Ppl
ENSRNOG00000016756	343.893688	1.86975172	Ptgir
ENSRNOG00000062101	427.84951	1.86997695	Ace
ENSRNOG00000003687	571.813837	1.87004484	Rgs2
ENSRNOG00000007415	260.766976	1.88045441	Ptgs1
ENSRNOG00000017136	229.901483	1.9032718	Syt17
ENSRNOG00000020552	215.972854	1.9090321	Fosl1
ENSRNOG00000005708	590.478687	1.98979179	Mmp16
ENSRNOG00000014363	565.599342	2.00268607	Arhgef3
ENSRNOG00000004972	542.556829	2.00471131	Upp1
ENSRNOG00000023720	132.636091	2.02997683	Ntm
ENSRNOG00000009734	2868.90475	2.03516737	Akr1b8
ENSRNOG00000006735	1811.99159	2.03558987	Cdkn2b
ENSRNOG00000005185	117.77919	2.10664072	Nxph3
ENSRNOG00000020579	599.232024	2.12798746	Col7a1
ENSRNOG00000007989	476.579395	2.13581063	Chst1
ENSRNOG00000019587	4268.04711	2.14636305	Ptprn
ENSRNOG00000011631	818.915667	2.15307362	Fst
ENSRNOG00000018094	184.131615	2.16005587	Sv2c
ENSRNOG00000043098	278.497577	2.18592361	Mt2A
ENSRNOG00000008187	459.702904	2.18857958	Ubash3b
ENSRNOG00000010960	992.776666	2.19143293	Ankh
ENSRNOG00000009005	767.198758	2.2128068	Slco2a1
ENSRNOG00000014061	124.090217	2.30216419	Dusp5
ENSRNOG00000027024	887.042686	2.34612743	Rgs16
ENSRNOG00000048967	263.985315	2.38365836	LOC688459
ENSRNOG00000020792	938.466377	2.3908686	Etv4
ENSRNOG00000049028	197.803054	2.43511392	C5ar2
ENSRNOG00000059837	160.912697	2.45954771	Cdkn2a
ENSRNOG00000010797	425.429535	2.48373796	Esm1
ENSRNOG00000004276	775.619862	2.50081192	Itga3

ENSRNOG00000002653	158.454426	2.50204245	Kcnk2
ENSRNOG00000024728	460.595426	2.59493045	Arhgap22
ENSRNOG00000016456	225.855468	2.59708814	Il33
ENSRNOG00000016156	739.638532	2.6028402	Nptxr
ENSRNOG00000000488	5127.6332	2.66620748	Hmga1
ENSRNOG00000004956	318.236689	2.74516414	Jade2
ENSRNOG00000011300	188.825873	2.78618468	AABR07031193.1
ENSRNOG00000001158	842.982263	2.79672882	Abcg1
ENSRNOG00000023896	3437.60794	2.90114288	Dusp6
ENSRNOG00000042460	261.973209	2.93631116	Hmga2
ENSRNOG00000016267	416.418593	3.15071897	Chst15
ENSRNOG00000017484	1219.75904	3.27375228	Gja5
ENSRNOG00000020918	7372.65047	3.31953222	Ccnd1
ENSRNOG00000053766	887.999851	3.53289592	Ramp3
ENSRNOG00000019718	136.423388	3.6316174	Galnt15
ENSRNOG00000002202	111.903234	3.8874247	Sowahb
ENSRNOG00000008057	122.630153	4.14865608	Krt7
ENSRNOG00000012098	760.972584	4.83274774	Adcyap1r1
ENSRNOG00000003741	308.586835	5.74181837	Nptx1
